# Millet-based crop planting strategies in the Songhua River Region during the liaojin (907-1234 AD) dynasties: A case of the Luotong Mountain City site

**DOI:** 10.3389/fpls.2022.1046178

**Published:** 2022-11-23

**Authors:** Chun Yang, Lin Ban, Xiaohong Lv, Dong Li, Kun Xu, Xiuhua Gao, Chunxue Wang

**Affiliations:** ^1^Institute of Cultural Relics and Archaeology of Jilin Province, Changchun, China; ^2^Bioarchaeology Laboratory, Jilin University, Changchun, China; ^3^School of Archaeology, Jilin University, Changchun, China

**Keywords:** millet-based agriculture, northeast china, barnyard millet, flotation, dryland agriculture

## Abstract

**Introduction:**

Millet-based dryland agriculture is the traditional mode of agricultural cultivation in northern China and has been of great significance to the emergence and development of Chinese civilization. However, although they are both millet-based agricultural production methods, with various subtypes in different regions of northern China. In the Songhua River Region in northeast China, the ecological environment and abundant natural resources led to the slow development of agriculture, and it was only after the Liaojin Dynasties that a mature farming industry was formed.

**Material and Method:**

We used the plant flotation instrument to flotation the soil samples unearthed in the Luotong Mountain City, a Liaojin period site in Songhua River Region, northeast China, and collected the charred plant seeds. Then observing them with the electron microscope, we identified and counted the plant seeds in this site.

**Result:**

It was found that this region is still a millet-based crop utilization structure, and a total of 11 types of charred agricultural crop seeds were excavated from flotation at the Luotong Mountain City site. And the barnyard millet crops occupy a prominent advantage, with ubiquity of more than 91%.

**Discussion:**

The ancestors of this region were still engaged in a millet-based agricultural strategy during this period, with a certain lag compared to the Central Plains’agricultural strategy where Triticeae crops had become dominant. In addition, the crop structure with the millet-based agriculture of the region is also somewhat different from that of the Central Plains. Through comparative studies of surrounding sites and reference to historical documents, it was found that this difference in crop structure is a phenomenon unique to the Songhua River Region and is related to the dietary habits of the local settled Jurchen nomads, who ate barnyard millet meal.

## Introduction

The development of settled agriculture contributed to the emergence of civilization. During the formation of human society, many plants have been domesticated, including rice, wheat, corn, millet, barley and many other food crops. However, due to geographical and climatic constraints, the domestication of plant species was different, and the pattern and extent of agricultural development were also unlike anywhere else in the world. As a result, different centres of agricultural origin have arisen around the world, one of which is East Asia (Crawford, 2008). In East Asia, China is the most important agricultural region.

Several studies have shown that China has a long history of plant domestication. Approximately 10,000 years ago, the beginnings of plant domestication arose in the northern and southern regions of China at almost the same time. More than 20 charred rice grains (*Oryza sativa*) were found at the Shangshan site in Zhejiang Province (Zhao and Jiang, 2016), and some broomcorn millet seeds (*Panicum miliaceum*) and foxtail millet seeds (*Setaria italica*) were found at the Donghulin site in Beijing (Zhao et al., 2020). During this period, the complete agricultural production mode had not yet been formed, but the ancient people had completed the initial domestication of plants (Zhao, 2014).

Agriculture originated from two separate routes of development, namely, rice farming in the south and dryland farming in the north (Liu, 2022). Approximately 8,000~6,000 years ago, rice grains were found in southern archaeological sites such as the Jiahu site, Henan Province (Zhang et al., 2018), the Kuohuqiao site in Zhejiang Province (Zheng et al., 2004), and the Hemudu site, Zhejiang Province (Natural History Section & Chekiang Provincial Museum, 1978). In contrast, millet crops have been found at sites in the north, such as the Cishan site in Hebei Province (Tong, 1984), Xinglonggou in Inner Mongolia Province (Sun, 2014), and Xinle in Liaoning Province (Wang and Pan, 1983). The plant remains found in this period are more abundant than those from the previous phase, but the agricultural production model had still not been formed (Zhao, 2014).

Approximately 5,000 years ago, systematic agricultural production took shape in the northern and southern regions of China. The Liangzhu site in Zhejiang Province in southern China (Zhao, 2010) unearthed a large number of rice remains and paddy fields, showing a highly developed agricultural civilisation, a state of affairs that proves that rice farming was fully established in the southern region. This mode of agriculture has continued into later times. Likewise, large quantities of foxtail millet and broomcorn millet seeds were found in many archaeological sites in northern China approximately 6500 years ago (Zhao, 2017; Fan et al., 2017; Cheng et al., 2022). A well-developed agricultural civilization was found at the Banpo site in Shaanxi (Institute of Archaeology of Chinese Academy of Sciences & Xi’an Banpo Museum of Shaanxi Province, 1963). This evidence marked the formation of dryland farming in northern China. Unlike the development of agriculture in southern China, however, the introduction of wheat (*Triticum aestivum*) to northern China approximately 4000 years ago led to a transformation of this major crop (Jin, 2021; Xu and Wang, 2018). As a result, the agricultural pattern of “southern rice and northern wheat” eventually formed in ancient China (Zhao, 2009).

However, although the millet crop was replaced by wheat at a later stage of agricultural development in northern China, its role as a native crop cannot be underestimated in the origin and development of early Chinese civilization. According to the studies, there is a continuous and stable history of domestication of the millet crop in the North China Plain and the Western Liaoning River Plain (Ma, 2014), but the situation of millet crop farming in some parts of northern China remains somewhat less clear in ancient China. The Songhua River Region (hereinafter SRR), another major cultural region in Northeast China besides the Western Liaoning River Plain, has been dominated by fishing and gathering as the economic model in the Neolithic period (Yuan, 2016). And the farming is also less developed, slightly different from the traditional Central Plains region. Therefore, in this case, even though the SRR eventually developed millet crop farming, there is still some discrepancy compared with the North China Plain and the Liaoning Plain area.

To clarify this discrepancy, we identified and counted the plant remains excavated by flotation in 2009 from the Luotong Mountain City (hereinafter LMC) site (Baidu Map: N42.3516; E125.99984) in the SRR and studied the agricultural use patterns in this region.

## Background

### Utilization of barnyard millet in China

Barnyard millet is an atypical agricultural crop that originated in East Asia. The earliest domestication of barnyard millet (*Echinochloa utilis*) as a food crop occurred in Japan, where it is known as “Japanese millet” (Crawford and Takamiya, 2008). The remains of cultivated barnyard millet have been found in many sites of the Jomon period in Japan. (Crawford, 2008). The cultivation and use of barnyard millet in Japan has a long history, and the Japanese still eat cultivated barnyard millet until modern times (Kumagai et al., 2011).

However, in the early domestication of agriculture in East Asia, barnyard millet was not as important as foxtail and broomcorn millet. Their domestication was regional and less common than that of the other two millet crops. After the historical period, with cultural exchange and social progress, the variety of crops available increased, and the barnyard millet became increasingly marginalized as a functional food. Thus, in the entire East Asian region during the historical period, only Japan had multiple uses for barnyard millets (Crawford, 2008).

In China, barnyard millet has suffered from a cold shoulder. In ancient China, barnyard millet was consumed only at certain times. Most of the time, their close relatives, the undomesticated barnyard grasses (*Echinochloa crusgalli*), are more frequently seen in archaeological sites. The earliest charred wild barnyard grass seeds have been found by Chinese researchers at the Shizitan Palaeolithic site (13,800 and 8,500 cal. BP) in Shanxi (Li, 2015). In addition, these wild seed remains were also found in Neolithic sites, such as the Baligang site (Deng and Gao, 2012), Shangshan site (Zhao and Jiang, 2016), and Beiqian site (Zhao, 2009). At the above mentioned archaeological sites in China, charred barnyard grass seeds were found sporadically, and their particles were small, which is quite different from the cultivated barnyard millet seen in Japan (Yang et al., 2010).

Although the archaeological evidence in prehistory does not provide evidence of the use of cultivated barnyard millet by the Chinese, ancient Chinese agricultural books do record some information. *Fan Sheng-Chih Shu (Fan Shengzhi’s Book on Agriculture)* wrote during the Western Han Dynasty (206 BC-8 AD) about the planting advantages and economic value of barnyard millet as a food crop, “*Barnyard millet can be grown in both flooded and dry conditions, and as long as it is planted, it will be harvested. It is also particularly luxuriant and suitable for planting in preparation for famine. The seeds of barnyard millet have grain, and when mature, they can be pounded out and cooked as barnyard meals, which is no worse than sorghum meal. It can also be used to make Baijiu (A Chinese alcoholic beverage made from grain)*” (Shi and Fan, 1959).

Jia Sixie of the Northern Wei Dynasty (386-534 AD) added, in his agriculture book, *Qimin Yaoshu (Essential techniques [or arts] for the common people)*, that *“Barnyard millets can be used to make Baijiu, and the smell is both fragrant and strong, much more than broomcorn millet wine and sorghum wine. Emperor Wudi of Wei Dynasty (155-220 AD) once ordered the chief agricultural officer to plant barnyard millets … The grains of barnyard millet could be ground into flour to feed the hungry in bad years; in good years, it could be used to feed cattle, horses, pigs and sheep”* (Cynthia et al., 2015).

In the Western Han Dynasty, ancient Chinese farmers already had mature knowledge of the barnyard millet and sometimes used it as human food. By the Wei Jin Dynasties (220-420 AD), the value of the barnyard millet as a brewing crop and livestock feed was more valued, and it was only consumed as human food during some famine periods. However, after the Northern Wei Dynasty, there are no more records of barnyard millets in agricultural books. It is clear that after the Northern Wei Dynasty barnyard millets gradually left the Chinese table.

### Agricultural conditions in Northeast China during the Liaojin Dynasties (907-1234 AD)

Northeast China, with its rich natural environment, has been of great importance and is one of the birthplaces of Chinese civilization. Since the Neolithic period, it has been an important settlement for nomadic fishing and hunting crowds and has developed a different form of civilization than that of the Central Plains. Before the establishment of the Liao Dynasty, the region was dominated by non-agricultural production methods (Han, 2021). Therefore, there was still a certain lag in the domestication and utilization pattern of crops in Northeast China during the Liaojin Dynasties compared with that in the Central Plains. Moreover, the agricultural development in the whole Northeast Chinese region was not the same during this period, with the agricultural situation in the Liaohe River basin area superior to that in the SRR (Ma, 2010).

However, the Liaojin Dynasties were also a period when agriculture flourished in north-eastern China. According to historical records, large-scale population migration occurred in the northeast during the Liaojin Dynasties. During the period when the Liao Kingdom and Jin Kingdom were confronted, one after another, with the Song Kingdom, the cultural exchange between the two sides also intensified. At the same time, the Liao and Jin governments realized the importance of agriculture and moved a large number of Han Chinese and Bohai people to the SRR to strengthen the development of agriculture in this region, which brought advanced farming techniques and abundant food crops to the region, resulting in a highly developed agricultural process (Han, 2021). In this historical context, the excavated sites of agricultural evidence in Northeast China, especially in the SRR, have become particularly important. These agricultural sites can give a glimpse of the interrelationship between nomadic fishing hunting and sedentary agriculture throughout Northeast China during the historical period and provides information on the advancement of livelihood patterns from nomadic pastoralism to highly developed farming in Northeast China (Zhao, 2020).

### The LMC site

**Figure 1:**
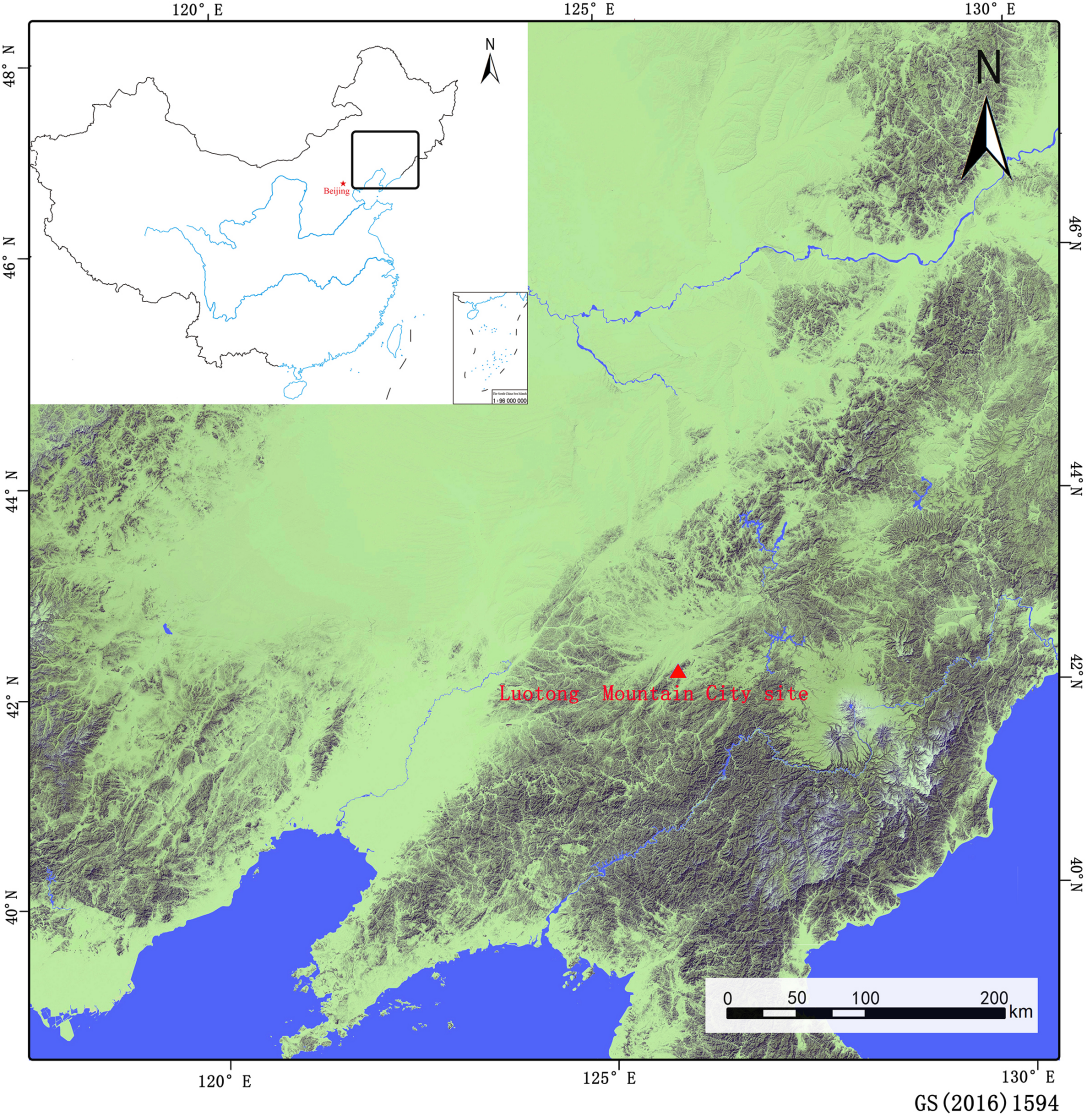
Location of the Loutong Mountain City site (Original Image Source: http://bzdt.ch.mnr.gov.cn and https://map.qq.com/).

The LMC site is located at the intersection of Liuhe County, Huinan County and Meihekou County in Jilin Province, between 125°17′ ~126°34′ east longitude and 41°53′~42°35′ north latitude (**Figure 1**). The mountainous area belongs to the Luotong Mountain Range of the Changbai Mountain Chain. The highest elevation reaches 1090 metres, with complex and treacherous terrain. The City was built in accordance with the mountain on the main peak of 960 metres above sea level in the central part of Luotong Mountain. Based on its form, construction techniques and geographical location, the site was presumed to have been built during the Goguryeo period of the Wei and Jin Dynasties (220~589 AD) and was an important military fortress for the northwards expansion of the ancient capital Gungnae City and Wandu Mountain City. It plays an important role in the study of the architecture of the ancient city of Goguryeo, the geography of the territory, military defence, and the development of the country’s politics, economy, culture, and military (Xu et al., 1985).

In addition, researchers have found that the site contains not only historical relics from the Goguryeo period, but also remains from the Liaojin Dynasties and even unearthed copper coins from the Song dynasty. It is clear that the city continued to be used for some time after the fall of Goguryeo and had close ties with the Central Plains. The discovery of this site is valuable not only for the study of Goguryeo history, but also for the transformation of the business model, ethnic integration and cultural exchange in northeast China.

## Materials and methods

From 2006 to 2009, the Institute of Cultural Relics and Archaeology of Jilin Province organized relevant personnel to systematically conduct a survey and three excavations on the LMC site. During the excavation work, the unearthed artefacts included ironworks, potteries, porcelain, jade artefacts, etc. The ironworks included arrowheads, spear blades, chisels, armor pieces, pots and pans. The pottery included pots, jars, and bowls; the jade includes rings and beads; and the porcelain included bottles. It is a very typical ancient Chinese city site (Xu et al., 1985). The materials used in this paper included charred plant remains belonging to the Liaojin Dynasties excavated from some important units in 2009.

### Sampling method

The archaeological excavation area was small, so the diversity of the collected flotation samples was somewhat limited. Therefore, a directional sampling method was chosen to sample a total of 13 house sites, one city gate and one drainage ditch excavated from the Liaojin Dynasties in the LMC site. Among them, 13 house sites of the Liaojin Dynasties were identified as the focus of sampling. Due to the shallow burial of the relics, which were quite close to the surface, there is a higher possibility of late human interference, and in consideration of maintaining the purity of the sample collection, no sampling was conducted on the stratum within the rectangular unit. After counting, the total number of samples collected by flotation reached 23, with a total of 337 L of soil samples and an average soil volume of 15 L per sample.

### Flotation

The samples collected from this excavation were separated in the field using a water wave flotation device (Crawford, 1984) and an 80 mesh sieve (0.2 mm pore size) for the collection of charred plant seeds. The floated plant seeds were dried in the shade and then identified and analysed in the phyto-archaeological laboratory. Ancient agricultural production was explored through flotation results mainly through the quantitative analysis of crop seed remains, but the use of flotation to obtain plant remains is subject to biases in absolute numbers (Zhao, 2004). Therefore, we later corrected this bias by combining it with the ubiquity statistics method. Ultimately, charred plant seed remains were obtained from all soil samples collected during flotation. After laboratory identification, these charred plant seed remains can be divided into two categories, charred wood chips and charred plant seeds. Plant seeds are the focus of this paper.

## Results

### Identification

#### Charred wood chips

Charred wood chips are the remnants of wood that has been burned, the main source of which is fuel, wood buildings, and wood that has been burned for other purposes. The total amount of charred wood chips obtained from flotation at the LMC site was 125.74 g through screening using a 1 mm pore size splitting sieve. The average amount of charred wood chips per sample reached 5.47 g (**Table 1**). This identification result of charred wood chips from the LMC site has a high content ratio compared with similar sites in the past. Considering that the total number of samples collected by flotation was small and the sampling context was relatively homogeneous, this high figure is likely to be a coincidence and not representative of the overall burial of charred wood chips in the Liaojin Dynasties cultural layer at the LMC site. In addition, the location of the site was conducive to the collection and use of timber, and it is probably for this reason that has a high ratio of charred wood chips.

**Table 1:** Statistics of charred wood chips from flotation samples in 2009 at the LMC site.

Sampling units	Sample quantity	Total weight (g)	Average weight(g)
House sites	18	157.83	8.77
City gate	3	4.03	1.34
Drainage ditch	1	0.863	0.863

#### Charred plant seeds

A total of 6,715 charred plant seeds were found in the 23 flotation samples collected from the site of LMC site (**Table 2**). The seed emergence rate of charred plants at the site was approximately 201 seeds/10 litres. Similar to the statistics of charred wood chips excavated from this site, this figure is significantly high in comparison with other sites. The charred plant seed species identified by microscopic observation included foxtail millet (*Setaria italica*), broomcorn millet (*Panicum miliaceum*), barnyard millet (*Echinochloa esculenta*), sorghum (*Sorghum bicolor*), barley (*Hordeum uhulgare*), oats (*Avena sativa*), buckwheat (*Fagopyrum esculentum*), soybean (*Glycine max*), cowpea (*Vigna*), bristlegrass *(Setaria*), grass family (*Poaceae*), legumes (*Leguminosae*), polygonum (*Polygonaceae*), Euphorbiaceae (*Euphorbiaceae*), cannabis (*Cannabis Sativa*), Perilla (*Perilla frutescens*), raspberry (*Rubus*), Huangbai (*Phellodendron chinense*), chenopodium (*Chenopodium*), cocklebur (*Xanthium sibiricum*), elderberry (*Sambucus chinensis*), kochia scoparia (*Kochia scoparia*), etc. In addition, there were a small number of unknown species of plant seeds that were not clearly characterized or had lost their key characteristic parts for identification due to more severe charring, so the species could not be accurately identified. The number of seeds of charred plants excavated is shown in **Table 2**.

**Table 2:** Statistics of plant seeds unearthed in 2009 at the LMC site.

Family	Genus/Species	Counts	Percentage
Poaceae	*Setaria italica*	2530	37.68%
	*Panicum miliaceum*	616	9.17%
	*Echinochloa esculenta*	2963	44.13%
	*Sorghum bicolor*	53	0.79%
	*Hordeum vulgare*	16	0.24%
	*Avena sativa*	120	1.79%
	*Setaria* sp.	243	3.62%
	*Panicum* sp.	10	0.15%
	Poaceae	1	0.01%
Polygonaceae	*Fagopyrum esculentum*	4	0.06%
	Polygonaceae	20	0.30%
Leguminosae	*Glycine max*	53	0.79%
	*Vigna unguiculata*	26	0.39%
	Leguminosae	5	0.07%
Euphorbiaceae	*Euphorbiaceae*	2	0.03%
Cannabaceae	*Cannabis Sativa*	1	0.01%
Lamiaceae	*Perilla frutescens*	12	0.18%
	*Amethystea caerulea*	5	0.07%
Rosaceae	*Rubus* sp.	4	0.06%
Rutaceae	*Phellodendron chinense*	1	0.01%
Chenopodiaceae	*Chenopodium* sp.	14	0.21%
Asteraceae	*Xanthium sibiricum*	1	0.01%
Adoxaceae	*Sambucus chinensis*	4	0.06%
Amaranthaceae	*Kochia scoparia*	11	0.16%
		6715	100.00%

### Charred agricultural crop seeds in Loutong Mountain City

**Figure 2:**
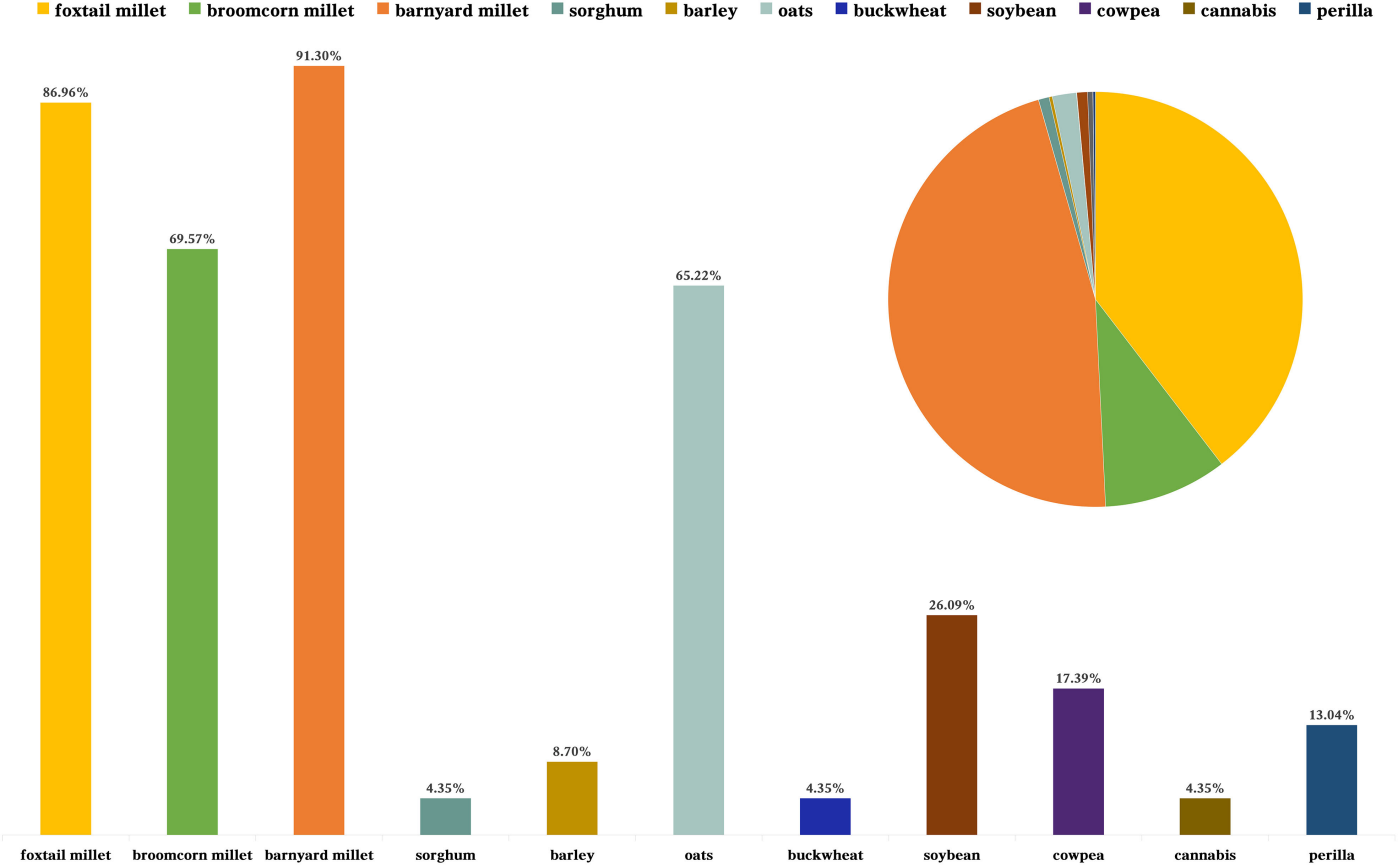
Statistical chart of the absolute number and ubiquity of agricultural crops at the LMC site.

**Figure 3:**
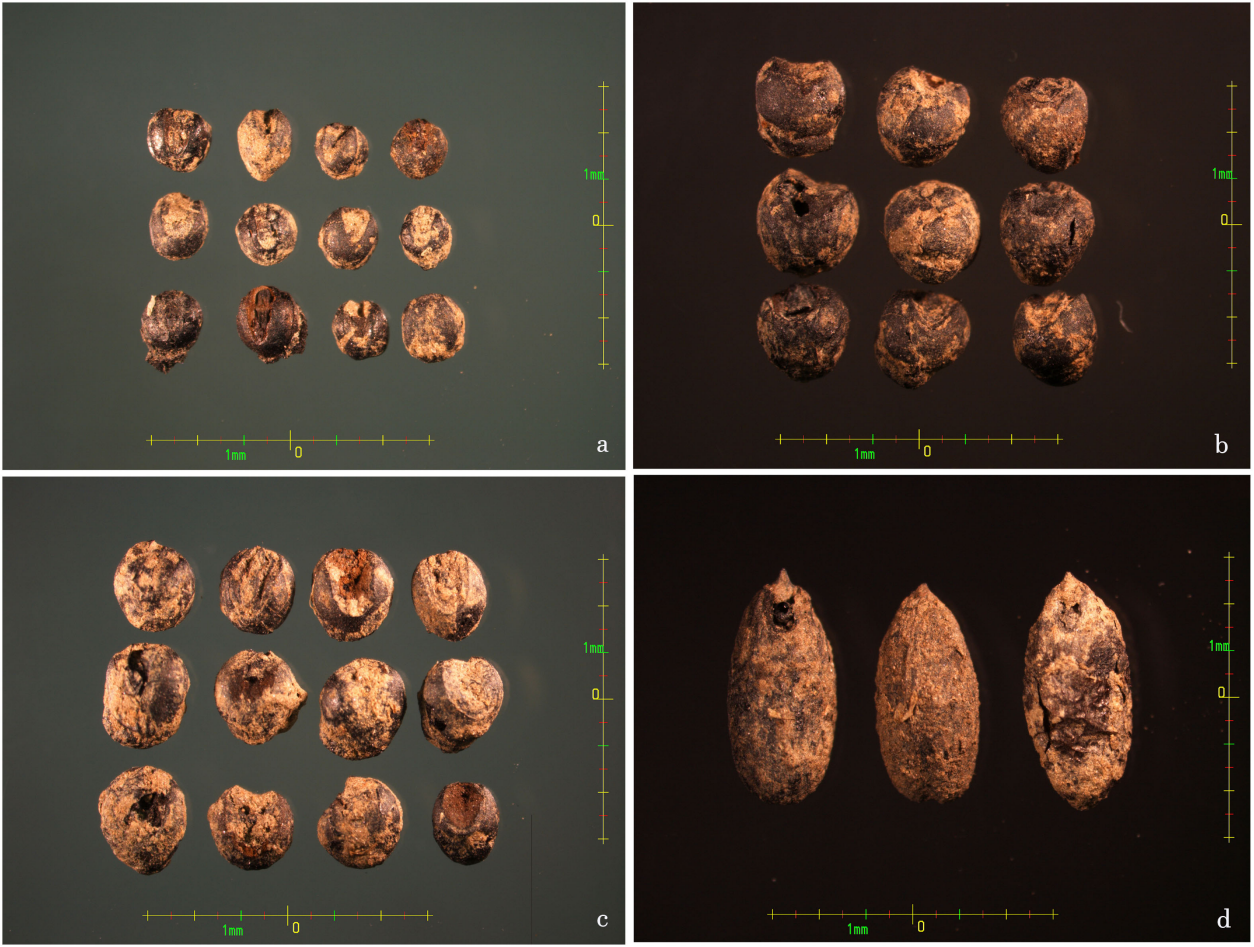
Images of LMC site plant seeds remain unearthed in part. **(A)** Charred foxtail millet seeds; **(B)** Charred broomcorn millet seeds; **(C)** Charred barnyard millet seeds; **(D)** Charred barley seeds.

A total of 11 types of charred agricultural crop seeds (**Figure 2**) were excavated from flotation at the LMC site. Among the 11 types of crop seeds, three types, foxtail millet, broomcorn millet and cultivated barnyard millet (**Figure 3**), showed a significant numerical advantage. These three crops are all millet crops, and all belong to the C4 plant. This result reflects that the agricultural production of the inhabitants of LMC during the Liaojin Dynasties was mainly based on the cultivation of millet crops, in line with the characteristics of dry farming in northern China. However, according to the difference in the number of charred crop seeds excavated, these three types of millet crops have different positions in the crop structure of the site.

The absolute number and ubiquity of cultivated barnyard millet occupy the first place, 46.70% and 91.30% (**Table 3**), respectively. It outnumbered the two typical northern crops in the traditional sense, foxtail millet and broomcorn millet, and was the most closely related grain to people’s lives at that time. The crop second only to the cultivated barnyard millet is foxtail millet, and its absolute number and ubiquity are close to the former, reaching 39.9% and 86.96% (**Table 3**), respectively, the first choice of food for the ancestors except for the cultivated barnyard millet. Broomcorn millet also occupies a certain proportion of the plant remains excavated at the site, with an absolute number and ubiquity of 9.71% and 69.57% (**Table 3**), respectively. Compared with the cultivated barnyard millet and foxtail millet, the quantity of broomcorn millet is much smaller, but its ubiquity is still higher. Therefore, it is assumed that, although millet is not as important as barnyard millet and foxtail millet, it was still the main food of the ancestors of LMC at that time, and it was an important food source for the ancestors in agricultural life.

**Table 3:** The absolute number and ubiquity of agricultural crops at the LMC site.

Common Name	Absolute Quantity	Absolute Quantity Ratio	Ubiquity
foxtail millet	2530	39.58%	86.96%
broomcorn millet	616	9.64%	69.57%
barnyard millet	2963	46.35%	91.30%
sorghum	53	0.83%	4.35%
barley	16	0.25%	8.70%
oats	120	1.88%	65.22%
buckwheat	2	0.03%	4.35%
soybean	53	0.83%	26.09%
cowpea	26	0.41%	17.39%
cannabis	1	0.02%	4.35%
perilla	12	0.19%	13.04%
Total	6392	100%	

The flotation samples from LMC site were also found to contain 53 grains of sorghum, with an ubiquity of 4.35% (**Table 3**). Despite the small number of discoveries, the flotation results of sorghum prove that it appeared on people’s tables in the SRR during the Liao and Jin Dynasties. Other grain crops, such as barley (**Figure 3**) and oats, were also found at the LMC site, but the number of these was slightly smaller and their proportion in the ubiquity was also low, which indicates that wheat crops were not the main crop in the production of crops during the Liaojin Dynasties at the LMC site, but there were abundant varieties.

**Figure 4:**
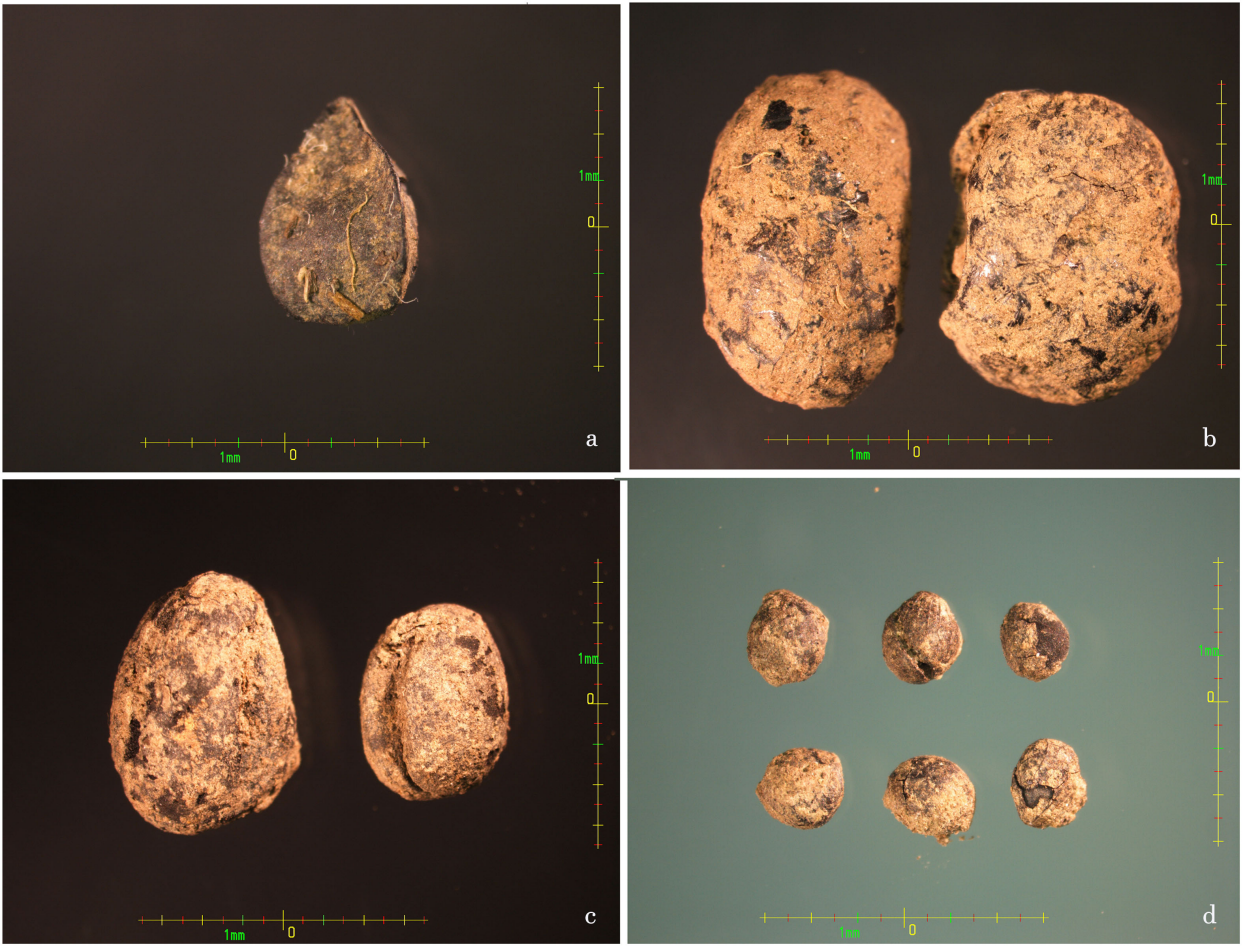
Images of LMC site plant seeds remain unearthed in part. **(A)** Charred buckwheat seeds; **(B)** Charred soybean seeds; **(C)** Charred cowpea seeds; **(D)** Charred perilla seeds.

In addition to the above species, crops such as soybean, cowpea, perilla (**Figure 4**), and cannabis are also found in the LMC site, but their quantities are not outstanding. Considering that they are not major food crops, it is not difficult to understand that their quantity ratio is lower than that of several kinds of millet.

## Discussion

### Barnyard millet in the SRR

The remains of millet crops excavated from the LMC site are still dominant, and this region also belongs to the millet-based dryland agriculture of Northern China. However, among the three types of foxtail millet, broomcorn millet and barnyard millet, barnyard millet is the most abundant, with an ubiquity of 91.3%. Whereas in most of China, barnyard millet is considered an agricultural pest grass, the excavation of such a large amount of barnyard millet from the Liaojin Dynasties LMC site is obviously different from the utilization of barnyard millet in most of China.

**Figure 5:**
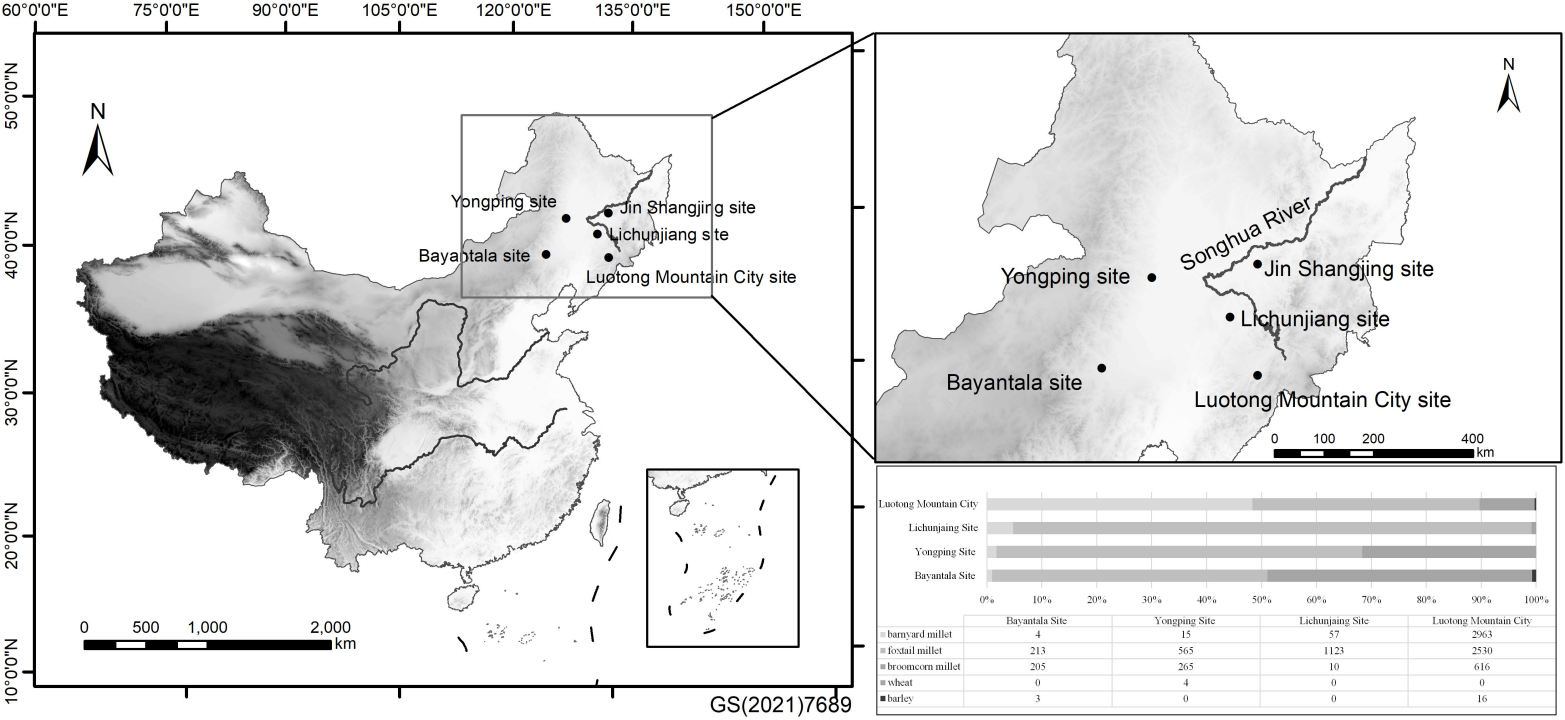
Geographical location and main crop proportions of archaeological sites of the Liaojin Dynasties in the SRR (a report on the flotation plant remains from Jinshangjing site has not yet been published). (Original Image Source: http://bzdt.ch.mnr.gov.cn).

After collecting data from other sites in the surrounding area, we found that the above phenomenon is not an exception. In Liaojin Dynasties sites such as the Jin Shangjing site (Baidu Map: N45.49388; E126.97093) in Heilongjiang, Bayantala site (Baidu Map: N43.36832; E119.88779) in Inner Mongolia (Sun, 2013), Lichunjiang site (Baidu Map: N44.168109; E125.394443) (Liang et al., 2009; Yang et al., 2010) and Yongping site (Baidu Map: N45.72348; E122.52983) in Jilin (Yang, 2014), a certain amount of charred cultivated barnyard millet seeds remained, although the amount of cultivated barnyard millet excavated from the sites was not as abundant as that of LMC. These sites are all concentrated in the SRR except for the Bayantala site (**Figure 5**). Therefore, dry farming in the SRR during the Liaojin Dynasties should have been different from that in the Central Plains and was a kind of regional millet farming.

This phenomenon is also consistent with ancient Chinese historical documents. The Song ambassador Ma Kuo in the *Mao Zhai Zi Xu (Autobiography in Maozhai)* recorded his own mission to the Jin kingdom about the food situation: “*From Xianzhou (咸州) to the area north of the Songhua River, people do not grow common millets, all they grow are barnyard millets, which are pounded into grain and made into barnyard millet meal. Once I saw the Jin emperor (Wan-yen A-ku-ta) and the chiefs of the Jin country eat together, and put a low table on the kang (a heatable brick bed in North China). Each person holds a bowl of barnyard millet meal, which is the so-called Jin kingdom royal banquet.”* (Zhao, 2005)

Song dynasty man Hong Jin also recorded the life of the Song royal family after the captivity by the Jin kingdom in *“Rong Zhai San Bi”(Rongzhai Essay)*, which also mentioned the barnyard millet: “*Since the change of Jingkang in the Northern Song Dynasty, all the people who were captured by the Jin Kingdom, regardless of the royals, or the officials of government, all of them were relegated to slavery and driven into servitude by the Jin Kingdom. Each person was given five Dou of barnyard millets (unhusked) in a month, and they were asked to pound the barnyard millets themselves to get one Dou and eight litres of the grains of barnyard millet as a month’s ration”* (Hong, 2019)*. “LiaoShi”(The History of Liao) also* recorded the scene of the Liao royal sacrifice using barnyard: “*One day, the shaman inserted a willow branch (a ritual practice) in the southeast of the canopy and toasted it with wine, broomcorn millets and barnyard millets to pray for blessings*” (Tuo, 1974).

It can be found through archaeological evidence and historical materials that in the Liaojin Dynasties, the Jurchen tribes in the SRR of northeast China would have had a dietary tradition different from that of the Han Chinese in the Central Plains, including eating barnyard millet meals.

This eating habit existed among both nobles and commoners, and was a relatively stable dietary tradition. We speculate that there are several possibilities about the formation of this tradition. (1) The high latitude and cold climate of northeastern China limited the types of crops suitable for cultivation, so the barnyard millet became the primary choice of the Jurchen people because of their low requirements for the growing environment. (2) The rich plant and animal resources and relatively sparse population in the SRR made the relationship between people and land harmonious. In this environmental context, inhabitants did not need to vigorously cultivate crops and develop agriculture to obtain living resources. Therefore, the barnyard millet is already sufficient to meet the inhabitants’ food crop needs. (3) This tradition may also be related to the Goguryeo that once settled in the area. However, the relationship between the two is not yet clear due to the limitations of excavated materials.

Combining the research results of the surrounding sites, we argue that eating barnyard millet meals tradition of Jurchen in the SRR is the result of a combination of the above three reasons. At the same time, this dietary tradition is strongly associated with Jurchen people, even with the expansion of the dominion of the Jurchen people, affecting areas further south (Shang et al., 2016). However, some ruling policies in the Liao Dynasty caused its decline.

The government of the Liao Dynasty implemented an immigration policy to promote agriculture in the region, moving agricultural people from the Central Plains and the Bohai Kingdom into the SRR. And probably under the effect of migration of the agricultural population, the dietary tradition of eating barnyard millet meals was also declined. The differences in the number of charred barnyard millet seeds excavated from different archaeological sites in the region reflect the changes on dietary traditions. And because of its remote location and the fact that it was once a city of Goguryeo, LMC site has preserved the most primitive tradition of eating barnyard millet and has unearthed the largest number of charred barnyard millet seeds.

### Millet-based crop planting strategies in the SRR

At the LMC site, barnyard millet is the most abundant and is the main source of grain; foxtail millet is the next most abundant, and broomcorn millet accounts for the smallest proportion of these three crops. However, among the three sites of Bayantala, Yongping, and Lichunjiang, foxtail millet excavation was the most abundant and dominant, and although there is also a certain amount of barnyard millet, its quantity was less than that of the LMC site.

At the Bayantara site, the number of Charred foxtail millet and broomcorn millet plant seeds excavated was comparable, 213 and 205, respectively, and the number of barnyard millet was extremely small, with only 4 being observed; at the Yongping site, the number of foxtail millet was the most abundant at 565, followed by 265 for millet and only 15 for barnyard millet; at the Lichunjiang site, the number of foxtail millet excavated was absolutely dominant, 1123, followed by 57 for barnyard millet, and extremely small for broomcorn millet, with only 1 being observed. The comparative study with the surrounding sites of the same period, Bayantala site, Yongping site and Lichunjiang site, reveals that in these four sites during the Liaojin Dynasties in the SRR, millet crops were still predominant, with foxtail millet as the main crop, followed by broomcorn millet and barnyard millet.

Wheat crops are in stark contrast to millet crops. Only 16 barley seeds were unearthed at the LMC site, and no wheat was observed. This situation also occurs in the surrounding sites. At the Bayantala site, only 3 grains of barley were unearthed, and no wheat was found; at the Yongping site, only 4 seeds of wheat were found; and at the Lichunjiang site, no wheat crop was found. This shows that wheat crops were not widely promoted and used in the SRR during the Liaojin Dynasties.

In addition, Charred seeds of sorghum, buckwheat, oats, and soybean miscellaneous grains have been found at the LMC site. Sorghum was also found at the Lichunjiang and Yongping sites, with only individual seeds. Similarly, buckwheat was also found at the Yongping site and Bayantala site, and soybeans were only excavated at the Yongping site. Such miscellaneous grains in the SRR during the Liaojin Dynasties should also occupy a certain proportion of people’s preferred recipes.

Moreover, charred cannabis seeds were excavated from the Liaojin Dynasties LMC site, Yongping site and Bayantala site in the SRR, and the number of cannabis charred seeds excavated from the Bayantala site even reaching 167. From this, it can be inferred that cannabis, an economic crop, was already commonly cultivated in the SRR during the Liaojin Dynasties.

All of the above provides evidence for the speculation on crop utilization during the Liaojin Dynasties in the SRR. The region is still a millet-based agricultural utilization model, but the proportion of the three types of foxtail millet, broomcorn millet and barnyard millet, varies from site to site, probably due to regional dietary differences. Wheat was not yet commonly cultivated in the region during the Liaojin Dynasties, and only a few seeds have been unearthed sporadically, which has a certain lag compared with the Central Plains during the same period, proving that the SRR may have been a relatively closed agricultural production unit during the Liaojin Dynasties. However, the excavation of non-staple food crops such as sorghum, oat, buckwheat, barley and soybean, proved that the region had a rich variety of crops, and this diversity of crops was obviously the result of cultural exchange.

This contradictory situation corresponded to the stage of agricultural development in the region at that time. The Liaojin Dynasties was a key period in the transformation of the production pattern of the SRR. Before the Liaojin Dynasties, the SRR was dominated by agricultural production methods, and during the Liaojin Dynasties, agriculture developed rapidly under the influence of the government’s policy of vigorously developing agriculture. Against this background, a relatively independent, but inclusive, pattern of crop utilization emerged and is expressed in archaeological sites, such as the LMC site, Bayantala site, Yongping site, and Lichunjiang site.

## Conclusion

Based on the study of the flotation charred crop seed remains excavated from the LMC site, and combined with the charred crop seed remains excavated from surrounding sites of the same period, the following conclusions can be drawn:

1. The millet-based crop cultivation strategy is still the main strategy for agricultural production in the city and even in the region during the Liaojin Dynasties, supplemented by sorghum, buckwheat, oats and other miscellaneous cereal cultivation. Among the three millet crops, barnyard millet has a very important position. This strategy may be related to the superior natural environment of Northeast China and the social environment of mixed ethnic groups.
2. During the Liaojin Dynasties, the crop structure of the entire SRR showed a certain regional independence. However, within this seemingly relatively independent area, there are some differences between sites, showing different characteristics of agricultural production. This contradiction is due to the historical background of the migration of the agricultural population during the Liaojin Dynasties.

## Data availability statement

The raw data supporting the conclusions of this article will be made available by the authors, without undue reservation.

## Author contributions

CW, CY and LB designed the study. CY, CW, LB and XL conducted the study. CY, DL, KX and XG carried out the flotation. CY, LB and CW identified charred plant seeds. CW, CY and LB wrote the main manuscript text. CW, LB and XL prepared figures. CY and LB have contributed equally to this paper and are co-first authors of this paper. All authors contributed to the article and approved the submitted version.

## Funding

This work was supported by the Fundamental Research Funds for the Central Universities, “Migration, Cultural Integration and Environmental Adaptation of Ancient Populations along the Belt and Road Based on the Bioarchaeological Perspective” (No.2022CXTD17) and the Graduate Innovation Fund of Jilin University (No.2022219).

## Acknowledgments

We thank archaeological excavators at Institute of Cultural Relics and Archaeology of Jilin Province for their careful excavation work. We thank Qiang Hu from the Chinese Academy of Environmental Sciences for his help with the mapping. We also thank Shu Wang, Han Dong, Hongyu Zhang, Puyu Chen, Rongyan Li from the Bioarchaeological Laboratory of Jilin University for their help with this work. We are very grateful to the editor Jianping Zhang and two reviewers for reviewing the manuscript.

## Conflict of interest

The authors declare that the research was conducted in the absence of any commercial or financial relationships that could be construed as a potential conflict of interest.

## Publisher’s note

All claims expressed in this article are solely those of the authors and do not necessarily represent those of their affiliated organizations, or those of the publisher, the editors and the reviewers. Any product that may be evaluated in this article, or claim that may be made by its manufacturer, is not guaranteed or endorsed by the publisher.
